# Interaction between dyslipidaemia, oxidative stress and inflammatory response in patients with angiographically proven coronary artery disease

**DOI:** 10.5830/CVJA-2010-092

**Published:** 2012-02

**Authors:** D Tayal, B Goswami, M Chaudhary, V Mallika, S Tyagi

**Affiliations:** Department of Biochemistry, GB Pant Hospital, New Delhi, India; Department of Biochemistry, GB Pant Hospital, New Delhi, India; Department of Biochemistry, GB Pant Hospital, New Delhi, India; Department of Biochemistry, GB Pant Hospital, New Delhi, India; Department of Cardiology, GB Pant Hospital, New Delhi, India

**Keywords:** nitric oxide, ferritin, apolipoprotein B, coronary artery disease

## Abstract

**Introduction:**

Coronary artery disease (CAD) is emerging as the biggest killer of the 21st century. A number of theories have been postulated to explain the aetiology of atherosclerosis. The present study attempts to elucidate the interaction, if any, between inflammation, oxidative stress and dyslipidaemia in CAD.

**Methods:**

A total of 753 patients undergoing angiography were evaluated and 476 were included in the study. The parameters studied included complete lipid profile, and apolipoprotein B, ferritin and nitric oxide (NO) levels. Statistical analysis was carried out to determine the interrelationship between these parameters and the best predictor of CAD risk. Cut-off points were determined from the receiver operating characteristics curves, and the specificity, sensitivity, positive predictive value, negative predictive value, odds ratio and confidence intervals were calculated.

**Results:**

The levels of the parameters studied increased with the stenotic state and a positive correlation was observed between ferritin, NO and apolipoprotein B. NO emerged as the most reliable predictor of CAD, with an area under the curve of 0.992 and sensitivity and specificity of 97 and 98%, respectively.

**Conclusion:**

Environmental and genetic risk factors for CAD interact in a highly complex manner to initiate the atherosclerotic process. These risk factors should be considered mutually inclusive, not exclusive when devising pharmacological interventions, as multi-factorial risk management is the cornerstone of CAD management

## Abstract

Coronary artery disease (CAD) is the leading cause of death in India. Moreover, about 50% of coronary heart disease-related deaths in India occur in people younger than 70 years of age, compared with only 22% in the West.^1^ The WHO estimated that, over the next 10 years, India would lose 237 billion US dollars due to heart disease, stroke and diabetes mellitus.

Factors presumed to be responsible for the higher prevalence of CAD in India include increased central obesity, insulin resistance, higher prevalence of diabetes mellitus and atherogenic dyslipidaemia, characterised by low high-density lipid (HDL) concentrations, increased triglycerides (TG), small dense lowdensity lipoprotein cholesterol (LDL-C) and lipoprotein (a) [Lp (a)] concentrations.^2^ The established risk factors of CAD, such as age, gender, cholesterol, smoking, hypertension and impaired glucose tolerance do not account for its overall risk.

Coronary artery disease, a major cause of morbidity and mortality, has a multi-factorial aetiology and long pre-clinical phase that further complicates the timely identification of a population at risk and the elucidation of molecular mechanisms that lead to the pathogenesis of this condition. Whereas most clinicians previously regarded an atheroma as a bland lesion, the current notion that inflammation and immune response contribute to atherogenesis has garnered increased interest.^3^ A picture emerges of a chronic disease that from its origin to its ultimate complications involves inflammatory cells (T cells, monocytes, macrophages), inflammatory proteins [cytokines, chemokines, ferritin, C-reactive protein (CRP), etc] and inflammatory responses from vascular cells.^4^ The present understanding of atherosclerosis is that of a complex phenomenon that encompasses the intricate interplay of dyslipidaemia, oxidative stress and inflammation.

Serum ferritin level has been evaluated as a marker of inflammation in our study. Many studies suggest the ‘iron hypothesis’ is a link between iron storage and the development of coronary artery disease.^5^-^7^ According to these studies, ferritin may act as a pro-oxidant. Iron donates an electron to promote the formation of reactive oxygen species via the Fenton reaction and this causes oxidation of LDL, a trigger for the development of atherosclerotic CAD.^6^,^7^ Inflammation is associated with an abnormality in the redox state in the vasculature. Oxidants, as reactive oxygen species, release iron from ferritin either directly or through haeme oxygenase.^8^ This accelerates atherosclerosis via the stimulation of LDL oxidation.

Nitric oxide (NO) is universally accepted as an important regulator of vascular tone, capillary permeability and platelet adhesion and is involved in other cellular functions. Most of the cytotoxicity attributed to NO is due to peroxynitrite, produced from the diffusion-controlled reaction between NO and another free radical, the superoxide anion (ROS).^9^ Under pathophysiological conditions, ROS and NO react readily and therefore the half-life of bioactive NO is reduced and reactive nitrogen species, particularly dinitrogen trioxide and peroxy nitrite are generated, causing significant damage to cellular components and leading to chromosomal alterations, protein nitration, lipid peroxidation, subsequent cellular dysfunction and cell death.^10^

Quantification of apolipoproteins A and B provide a measure of the total number of anti-atherogenic and pro-atherogenic particles in the plasma. Apo-B-100 molecules present in lipoproteins lead to their entrapment in the arterial wall. Apo-B-100 are also taken up by macrophages in the arterial wall in native or oxidised form and orchestrate an inflammatory response in the arterial wall that promotes atherogenesis.^11^

Our study attempted to evaluate the interrelationship between dyslipidaemia, oxidative stress and inflammation in the aetiopathogenesis of atherosclerosis in angiographically proven CAD patents. This was accomplished by evaluation of the levels of the markers of dyslipidaemia, oxidative stress and inflammation; namely, apolipoprotein B (Apo B), NO and ferritin, respectively, in the study group.

## Methods

This study was conducted in the Department of Biochemistry in collaboration with the Department of Cardiology, Govind Ballabh Pant Hospital, New Delhi, India. A total of 753 patients who underwent coronary angiography were included in the study and after initial screening for exclusion criteria, 476 patients were finally enrolled.

Subjects were excluded from the study if they presented with congenital heart disease, valvular heart disease, cardiomyopathy and cardiogenic shock or were taking any nitrate preparation. Patients suffering from known liver, thyroid or renal disease, or malignancy, and those with acute viral infection within the previous four weeks were excluded. Patients with a history of recent use of oral contraceptives, anticonvulsants and retinoic acid were also excluded.

The number of significantly stenosed coronary arteries and lesions determined the severity of the coronary artery disease. Angiograms were assessed by the cardiologist, which enabled sub-classification of patients into four groups: G0 = no stenosis in any coronary vessel, G1 = one vessel stenosed, G2 = two vessels stenosed, with more than 50% stenosis, G3 = all three major vessels stenosed, with more than 50% stenosis.

Data on cardiovascular risk factors such as dyslipidaemia, diabetes mellitus, hypertension, smoking (currently or recently stopped) and medical treatment of all subjects were recorded. Ethical committee clearance was obtained before the commencement of the study. Informed consent was obtained from each patient according to the guidelines of the ethics committee.

## Analytical measurements

After overnight fasting and prior to coronary angiography, blood samples were drawn in a plain vacutainer under sterile conditions. The serum was separated and frozen at –50°C until further analysis. Serum ferritin was assayed using a solid-phase enzyme immunoassay based on the sandwich principle. The kit used for ferritin estimation was obtained from Syntron Bioresearch, Inc, USA.

Nitric oxide is a very unstable compound, therefore its level in the serum was determined indirectly by the measurement of its stable decomposition product, nitrite and nitrate, using the Griess reaction.^12^ This involves formation of a chromophore during the reaction of nitrite with sulphanilamide and N-(-naphthyl) ethylenediamine (Griess reagent) to produce a purple azo compound, which was measured photometerically at 543 nm.

Apolipoprotein B-100 levels were estimated using an immunoturbidimetry kit (Randox Laboratories Ltd, Antrium, UK) on an Olympus AU400 autoanalyser (Olympus, Germany). Total cholesterol, TG and HDL-C concentrations were quantified on an Olympus AU400 autoanalyser using standard kits. LDL-C was calculated using Friedwald’s formula: TC – (HDL-C + TG/5).^13^ The calculation of small dense LDL was done using the formula of Hattori *et al*.^14^

## Statistical analysis

Quantitative values of continuous variables were expressed as mean ± standard deviation. ANOVA was used to compare different parameters in the four groups. Pearson’s correlation was applied to test for associations between the continuous variables. The receiver operating characteristics (ROC) curves were plotted for NO, ferritin and apolipoprotein B and the area under the curve was calculated. Cut-off points were determined from the ROC curves and the specificity, sensitivity, positive predictive value, negative predictive value, odds ratio and confidence intervals were calculated. A two-tailed *p*-value < 0.05 was accepted as statistically significant for all the results. Statistical analysis was carried out using SPSS for windows 12.0 software (SPSS Inc, Chicago, IL, USA).

The positive predictive value, precision rate or post-test probability of disease is the proportion of patients with positive test results who are correctly diagnosed. The negative predictive value is the proportion of patients with negative test results who are correctly diagnosed.

The likelihood ratio incorporates both the sensitivity and specificity of the test and provides a direct estimate of how much a test result will change the odds of having a disease. The likelihood ratio for a positive result (LR+) indicates how much the odds of the disease increase when a test is positive. The likelihood ratio for a negative result (LR−) indicates how much the odds of the disease decrease when a test is negative. LR+ = true positives/false positives and LR− = false negatives/true negatives.

A likelihood ratio of > 1 indicates that the test result is associated with the disease. A likelihood ratio < 1 indicates that the result is associated with absence of the disease. Tests where the likelihood ratios lie close to 1 have little practical significance as the post-test probability (odds) is little different from the pre-test probability. When the positive likelihood ratio is > 5 or the negative likelihood ratio is < 0.2 (or 1/5) then it can be applied to the pre-test probability of a patient having the disease, in order to estimate a post-test probability of the disease state.

The odds ratio is a measure of effect size, describing the strength of association or non-independence between two binary data values.

## Results

Table 1 shows the demographics of the study population: 135 patients had single-vessel, 174 had double-vessel and 114 patients had triple-vessel stenosis. Fifty-three patients were categorised as without any stenosis. Risk-factor analysis showed that about 73 patients with triple-vessel disease were diabetic compared to only seven in the control group. Similarly, higher incidence of the other risk factors for CAD, namely smoking, hypertension and previous history of CAD was noticed in the patients with stenosis compared to those in the control group.

**Table 1:** Baseline Characteristics In All The Groups

*Parameter*	*Normal angiography (G0)*	*Single vessel (G1)*	*Double vessel (G2)*	*Triple vessel (G3)*
Number	53	135	174	114
Age (years)	54.3 ± 8.9	55.6 ± 9.5	55.8 ± 7.5	56.8 ± 8.6
Gender (M/F)	42/11	95/45	99/65	87/27
Diabetes (*n*)	7	34	59	73
Smoking (*n*)	8	28	40	55
Hypertension (*n*)	18	33	55	59
Previous H/O MI (*n*)	0	22	45	56

The levels of total cholesterol, triglycerides, LDL-C, ferritin, Apo B and nitric oxide were higher in the patients with stenosis compared to the controls. The levels were also higher in those with triple-vessel disease compared to those with single- and double-vessel disease (Table 2).

**Table 2:** Biochemical Parameters In Patients With Varying Levels Of Stenosis

*Parameter*	*No stenosis (G0)*	*Single-vessel stenosis (G1)*	*Double-vessel stenosis (G2)*	*Triple-vessel stenosis (G3)*
Total cholesterol (mg/dl)	143.6 ± 28.3	147.9 ± 28.6	154.7 ± 30.7^d^	175.9 ± 34.5^ce^
Triglyceride (mg/dl)	136.3 ± 42.4	147.7 ± 36.6	152.3 ± 35.5	148.7 ± 42.9
High-density lipoprotein (mg/dl)	36.8 ± 9.8	37.5 ± 9	35.0 ± 9.7	34.6 ± 6.7
Low-density lipoprotein (mg/dl)	80.7 ± 30.1	80.8 ± 27.9	88.9 ± 30.4	111.6 ± 33.2^cef^
Apolipoprotein B (mg/dl)	70.8 ± 17.1	91.8 ± 17.6^a^	97.4 ± 14.4^d^	148.1 ± 24.9^cef^
Small dense LDL (sdLDL)	1.85 ± 1.47	1.16 ± 0.65^a^	1.19 ± 0.62^d^	0.91 ± 0.38^f^
Nitric oxide (µmol/IU)	24.7 ± 2.2	34.1 ± 3.3^a^	48.7 ± 6.3^bd^	69.9 ± 5.7^cef^
Ferritin (ng/ml)	45 ± 21.2	93.4 ± 39.0^a^	177.8 ± 58^bd^	245.6 ± 40^cef^

Statistically significant difference between the groups is expressed as follows: ^a^G0 vs G1; ^b^G1 vs G2; ^c^G1 vs G3; ^d^G0 vs G1; ^e^G2 vs G3; ^f^G0 vs G1.

It can be inferred from Table 3 that dyslipidaemia, oxidative stress and inflammation interacted in the pathogenesis of CAD as total cholesterol, LDL-C and apo B, and they had a significant positive correlation with ferritin and NO. Among the markers of dyslipidaemia, Apo B had a very strong interrelationship with ferritin and NO (*r* = 0.709, *r* = 0.776, respectively). NO emerged as the best marker of CAD in our study, with an area under the curve of 0.992 compared to 0.901 and 0.955 for Apo B and ferritin, respectively (Table 4).

**Table 3:** Pearson’s Correlation Between The Dyslipidaemia, Inflammatory And Oxidative Stress Parameters

*Parameter*	R-*value*	p-*value*
Apolipoprotein B with ferritin	0.709	< 0.01
Apolipoprotein B with nitric oxide	0.776	< 0.01
Ferritin with nitric oxide	0.860	< 0.01
Cholesterol with ferritin	0.275	< 0.01
Cholesterol with nitric oxide	0.351	< 0.01
Low-density lipoprotein with feritin	0.267	< 0.01
Low-density lipoprotein with nitric oxide	0.358	< 0.01

**Table 4:** Comparison Of The Attributes Of The Various Parameters Under Study

	*Nitric oxide*	*Apolipoprotein B*	*Ferritin*
Area under curve	0.992	0.901	0.955
Sensitivity	97.3	88.9	92.3
Specificity	98	81.2	87.4
Positive likelihood ratio	49	4.72	7.34
Negative likelihood ratio	0.03	0.14	0.08
Positive predictive value	99.4	95.3	96.6
Negative predictive value	91.7	63.1	75.2
Odds ratio (95% CI)	158 (94.5–264.1)	44.1 (26.6–72.9)	81.5 (49.9–133)

Table 4 highlights the odds ratio and the predictive values of nitric oxide, ferritin and apolipoprotein B. It can be seen that the sensitivity and specificity of NO was highest compared to ferritin and apo B (97 and 98%; 92 and 87%; 89 and 81%, respectively). The superior predictive power of NO for the assessment of risk for CAD was also substantiated by the high positive predictive value, positive likelihood ratio and odds ratio compared to the other two parameters under study.

## Discussion

The prevalence of CAD in Asian Indians has been increasing rapidly and has reached alarming levels.^15^-^18^ It was the CAD-prone North Indian population that constituted our study population. A better appreciation of the atherogenic effects of well-known cardiovascular risk factors has been accompanied by understanding the sum of these factors, i.e. the global risk profile provides better predictive power than any single risk factor. In addition, a number of more recently identified and less well-known factors have received intense investigation over the past few years.^19^-^21^

The availability of effective therapies for preventing a first myocardial infarction renders imperative the need to identify individuals at risk, for concerted intervention, before problems manifest.^22^,^23^ In the present study, it was found that serum ferritin levels increased from those with normal coronary angiography to those with increased arterial stenotic states. These results are in accordance with those obtained in other studies, which favoured the ‘iron hypothesis’ in coronary artery disease.^5^-^7^ One of the studies^8^ demonstrated that protein expression of ferritin light chains increased 1.9-fold in diseased coronary arteries compared to normal ones.

A few studies have documented an increase in nitric oxide concentration in stenosed coronary vessels in comparison with normal vessels.^24^,^25^ In our study, the concentration of nitric oxide also increased in accordance with the level of stenosis. With ferritin being an inflammatory marker, we found a significant positive correlation between ferritin and nitric oxide levels. Stimulation of macrophages with interferon and lipopolysaccharides induced NO synthesis and activated iron regulatory protein (IRP) binding by IRP 1 and 2. The regulatory cross talk between iron metabolism and NO was further highlighted by the transcriptional regulation of the inducible NO synthase gene by iron.^26^ Our results confirm those of other studies, which showed that inflammation and inflammatory cytokines, which are known to stimulate NO production, stimulated ferritin synthesis.^27^

Another highlight of our study was detecting hyperapolipoproteinaemia in the CAD patients. The Quebec Heart study demonstrated accelerated CAD in patients with hyperapolipoproteinaemia B.^28^ In a study conducted by Genest *et al*. in 321 men, it was observed that the level of apolipoprotein B was increased and that of apolipoprotein A decreased in myocardial infarction patients.^29^ A highly significant positive correlation between ferritin and apolipoprotein B levels was observed in our study population. Apolipoprotein B levels signify the extent of dyslipidaemia. The positive correlation between these two parameters throws some light on the complex interaction between inflammation and dyslipidaemia in the pathogenesis of atherosclerosis and its ultimate clinical manifestation, namely acute coronary syndrome, which includes myocardial infarction.

The most typical change in lipoprotein metabolism during infection and inflammation is hypertriglyceridaemia. In addition to the observed changes in LDL-C levels, a change in LDL size and their susceptibility to oxidation was also noticed. Enhanced LDL oxidation has been documented during infection and inflammation. Moreover there were reductions in the plasma proteins proposed to play a major role in HDL metabolism and reverse cholesterol transport, including lecithin, cholesterol acyl transferase (LCAT), cholesterol ester transport protein (CETP), hepatic lipase (HL) and phospholipid transfer protein (PLTP). Because of these changes in HDL metabolism, it was postulated that the main function of HDL, namely its role in protecting LDL against oxidation and reverse cholesterol transport (RCT) may be decreased in acute-phase responses (APR).^30^,^31^

In the present study we found that dyslipidaemia correlated positively with NO concentration. Peroxynitrite, formed by the reaction of NO with free radicals, triggers lipid peroxidation in membranes^32^ and lipoproteins by removing a hydrogen atom from polyunsaturated fatty acids (PUFA), leading to the formation of lipid hydroperoxyradicals, conjugated dienes and aldehydes.^33^ One study demonstrated that in older patients, the immunoreactivity of NO was found in close association with foam cells, vascular endothelium and in the neo-intima of advanced atherosclerotic lesions.^34^

Peroxynitrite-modified LDL also binds with high affinity to scavenger receptors and leads to the accumulation of oxidised cholesteryl esters and foam cell formation, which is a key early event in atherogenesis.^35^,^36^ Another study demonstrated that reactive nitrogen species generated by the myeloperoxidase/hydrogen peroxide/nitrite system of monocytes converted LDL to a form (NO_2_-LDL) that was readily taken up and degraded by macrophages, leading to massive cholesterol deposition and foam cell formation.^37^

Atherosclerosis is now known to be associated with multiple aetiologies: oxidative stress, dyslipidaemia, inflammation and other novel risk factors. These interact at the molecular level in a highly complex manner to influence the entire process of atherogenesis.

The main limitation of our study was the lack of follow-up data due to lack of patient compliance and administrative constraints. A large-scale prospective study with follow-up data would facilitate our understanding of the present perspective on CAD in India.

## Conclusion

Our study highlights the interplay between ferritin, NO and apolipoprotein B levels. The ever-increasing knowledge on the various risk factors and molecular mechanisms underlying CAD calls for a critical appraisal of the existing understanding and shortcomings in order to better manage this pandemic.
